# User and staff experiences of a revised process for coordinating support with shared decision making in the comorbidity field of practice: a qualitative interview study

**DOI:** 10.1080/17482631.2024.2447095

**Published:** 2024-12-26

**Authors:** Amanda Jones

**Affiliations:** School of Education, Health and Social Studies, Dalarna University, Falun, Sweden

**Keywords:** Shared decision-making, social services, healthcare, substance use problems, mental health, qualitative interview study, thematic analysis

## Abstract

**Purpose:**

This study aims to explore user and staff experiences of a revised process for coordinated individual planning (CIP) that involves the user alongside staff from social services and healthcare and incorporates shared decision-making (SDM).

**Method:**

Eight staff members and five users participated in individual semi-structured interviews. The collected data were analysed using reflexive thematic analysis.

**Results:**

Users and staff experienced that the revised CIP process facilitates emotional security through predictability. This predictability is attributed to the predetermined structure in the CIP process and the user involvement enabled through preparations, as well as the mutual trust that arises from following through on expectations and commitments. Furthermore, the importance of partnerships was highlighted. This refers to the intention of respecting users as contributors, the joint understanding achieved between staff and users, and the continuity of care ensured through staff collaboration.

**Conclusion:**

The findings show that incorporating SDM through the CIP process can enhance structure, user involvement, and a sense of emotional security through the process.

## Introduction

Drawing on the principles of democracy and citizenship (Beresford & Croft, 2004; Vedung & Dahlberg, 2013), user participation has been highlighted as a priority in social work and healthcare across many countries. This emphasis is reflected in its integration into policies such as laws and ethical guidelines (Askheim et al., 2017; Eriksson, 2015; The Union for Professionals, 2017). For user participation to occur, users need to be heard, viewed upon as credible, and taken seriously (Kurs & Grinshpoon, 2018). Thus, users need to be perceived as reliable sources of knowledge and capable “epistemic subjects” (Fricker, 2018; Nouf & Ineland, 2023). Examples of reasons offered for viewing users as capable and credible include that they have a lived experience and knowledge that others lack (Jones et al., 2021; Laitila et al., 2011) and that the users can contribute with their valuable knowledge (Nouf & Ineland, 2023). However, previous research illustrates that users with substance use problems and mental illness (henceforth, comorbidity) share descriptions of not being heard, understood, or trusted in encounters with staff (Jones et al., 2021; Laitila et al., 2011). People with comorbidity are therefore subjected to *epistemic injustice* (Fricker, 2018) when they are dismissed as not being trustworthy knowledge bearers (Kurs & Grinshpoon, 2018; Nouf & Ineland, 2023).

An individual with comorbidity can need coordinated support from social services and healthcare. A decision-making process designed to support individuals in receiving coordinated support tailored to their needs and circumstances is coordinated individual planning (CIP). The decisions discussed in the CIP process can include medication, housing, financial support, work-related rehabilitation, and psychosocial support (Matscheck et al., 2019). Hence, there is a broad range of decisions, involving different units from social services and healthcare, which can be discussed and made in the CIP process. Previous research regarding user participation in CIP and similar interprofessional team meetings in the Nordic context illustrate both the importance of user involvement for a useful plan to be established (Beck et al., 2021), but also a lack of user participation (Matscheck & Piuva, 2022; Wenaas et al., 2021). Wenaas et al. (2021, p. 196) illustrated the *absence of user knowledge* in the coordination of support, both regarding staff not providing users with knowledge and also the devaluation of user knowledge compared to staff knowledge.

Shared decision-making (SDM) is an approach designed to enhance user participation by emphasizing that users are as valuable knowledge contributors as staff (Adams & Grieder, 2014; Knutsson & Schön, 2020). When utilizing SDM, users and staff share information and knowledge with each other and discuss different treatment options to facilitate collaboration in the decision-making process (Elwyn et al., 2012). Nykänen (2020) argues that the support for SDM to be used in social services is higher compared to other approaches. Moreover, it has been asserted that SDM can be used because of an ethical imperative (Hamann & Heres, 2019), and because SDM is in alignment with core values of the social work profession, such as equality and fairness in knowledge contributions between user and staff (Nykänen, 2020) and user autonomy (Lukens et al., 2013). There are different models of utilizing SDM in practice (Chmielowska et al., 2023). In the present study, a revised CIP process that involves SDM (Knutsson & Schön, 2020) has been explored, and the innovation is further described in the Method section.

To date, only one systematic review has investigated the utilization of SDM for individuals with comorbidity. Fisher et al. (2021) illustrate in a systematic review that SDM has a positive influence on user involvement in treatment decisions, with users having greater autonomy and a more active role in the decision-making process. However, regarding user symptom-related outcomes the findings are more ambiguous, and there are results showing both improved symptoms, such as better mental health and less drug use severity, but also no influence on quality of life, or even more days with alcohol consumption compared to usual care.

When utilized in a clinical mental health setting, staff experienced SDM as challenging in relation to the health conditions of the users. For example, staff perceived the insights of the users regarding their diagnosis to be limited. The staff mostly shared information regarding the diagnosis and health status of the users while providing less information regarding different treatment options (Haugom et al., 2020). Grim et al. (2019) also illustrate that staff share limited information regarding treatment options because they want to protect the user from self-destructive decisions. In a clinical mental health setting, users have experienced that SDM facilitates a collaborative decision-making between them and staff, in which both parties contribute with their expertise, fostering in the users a sense of individual recognition and comfort in engaging with the process. Information regarding what the decision might entail decreases the initial hesitance when the users are invited to be involved in the decision-making (Gibson et al., 2020).

More knowledge is needed regarding user participation in CIPs and how it can be strengthened (Matscheck & Piuva, 2022). Users with comorbidity risk being subjected to epistemic injustice (Nouf & Ineland, 2023), and strategies designed to strengthen users' positions as knowledge bearers are important to counteract this. SDM is such a method; however, much research regarding SDM within the mental health field focuses on medical decisions made in patient–doctor interactions, and it is suggested to look beyond this context when exploring SDM (Chmielowska et al., 2023). Hence, there is a paucity of knowledge regarding the use of SDM in other contexts, such as CIP, which involves a broader range of decisions than solely medical and several members of staff. Moreover, there is limited knowledge regarding how users with comorbidity and staff in these services experience SDM. For example, there is a paucity of knowledge regarding SDM in social work encounters (Nykänen et al., 2023; Rosenberg et al., 2017; Schön et al., 2018) and related to users with comorbidity Fisher et al. (2021).

The aim of the present study was to explore user and staff, in the comorbidity field of practice, experiences of a revised CIP process that incorporates SDM.

## Theoretical framework: epistemic (in)justice

Epistemic injustice is recognized as interactions in which individuals are not viewed upon as capable and credible epistemic subjects. One form of epistemic injustice is testimonial, meaning that a person is regarded as untrustworthy due to prejudice (Fricker, 2018). This prejudice can relate to belonging to a certain group (e.g., gender, ethnicity) (Fricker, 2018) or due to having a mental illness (Grim et al., 2019; Kurs & Grinshpoon, 2018; Nouf & Ineland, 2023).

There are also other concepts linked to epistemic (in)justice, such as active and passive participation (Nouf & Ineland, 2023). Active participation is understood as instances where users are viewed upon as valuable knowledge contributors that can initiate discussions, elaborate on their preferences, and reject staffs’ proposals, in contrast to passive participation in which users are foremost participants at a meeting due to routines and are expected to be compliant to staffs proposals (Nouf & Ineland, 2023).

## Methodology

To gain a deeper understanding of users and staff experiences of utilizing the revised CIP process, an exploratory qualitative interview study was conducted using a semi-structured interview guide. Both staff and users’ experiences were explored to gain a comprehensive understanding of the experiences of the revised CIP-process from both perspectives. Thematic analysis was used to analyse the meaning of the data across datasets.

### Context

The implementation of the revised CIP process that incorporates SDM was supported at three sites in the middle of Sweden. Each site involved staff from social services and healthcare. A study focusing on staff perspectives of the implementation process is reported elsewhere (Jones et al., in manuscript).

#### Innovation

**Figure 1.**
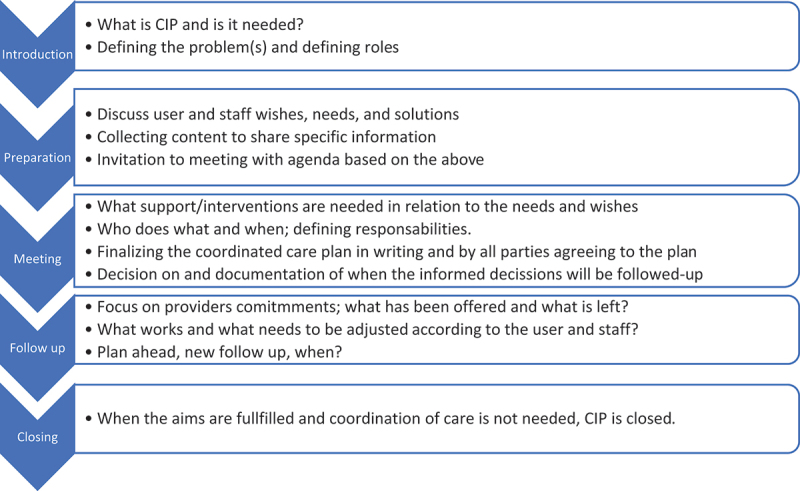
The five steps of the revised CIP process.

The innovation is a co-created process that integrates user participation through SDM in CIP, as described by Knutsson and Schön (2020). It represents an adaptation of how CIPs have traditionally been applied in practice, addressing areas such as the previously underemphasized preparation and follow-up phases. The primary adjustments focus on improving the preparation for CIP meetings to better support user involvement. This preparation involves discussions among staff members and between staff and the user. During the CIP meeting, it is emphasized that only information brought forward in the preparation phase is discussed. A follow-up meeting is scheduled during the CIP meeting, and during the follow-up, discussions are held regarding the fulfilment of goals and what additional support, if any, is needed. The five steps of the revised process are illustrated in Figure 1.

The core elements of this process are ongoing discussions among all parties, during which information and knowledge are shared over time on topics such as the user’s situation and different treatment options, in alignment with SDM. Furthermore, the process includes a new, co-created CIP form designed to support both the SDM discussions and the documentation of the CIP. For a detailed description of the development of the process and the CIP form, see Knutsson and Schön (2020).

### Recruitment

Recruitment was done through convenience sampling (Polit & Beck, 2021) by emailing staff at the three sites, who distributed information about the study to staff. Staff who had used the revised CIP process were encouraged to contact the author to participate in the study. Users who participated in the revised CIP process were informed about the study by staff. If the user wanted more information about the study, their contact details were shared with the author who thereafter contacted them. In the initial contact with the user, the authors received more information about the study and were asked if they wanted to participate. All users who were contacted by the author said yes to participation and times for interviews were booked.

The inclusion criteria for users alongside experience of the revised CIP process, were, 18–65 years of age, and had experienced-based knowledge regarding comorbidity. However, a formal diagnosis was not an inclusion criterion because the focus was on their experiences of their encounters with services, not on their problems per se. Hence, all users had experiences of contact with the social services, healthcare, and the revised CIP process.

Inclusion criteria for the staff included working within services that were a part of the implementation of the revised CIP process and having experienced the revised CIP process.

### Participants and data collection

A total of eight staff members and five users took part in individual interviews. Some of the participants had participated in the same CIP process (staff and users participating in the same CIP process together), whilst some of the staff were not connected to any of the users. All interviewed users were female. There have been a variety of previous experiences of CIP processes, from several to no previous CIP experience. Of the eight interviewed staff members, most worked within social services (*n* = 6) and most were female (*n* = 6). Staff had a variety of experiences of CIP prior to the implementation, from having participated in a few CIP meetings to participating in CIP on a weekly basis.

All interviews were held by telephone by the author and recorded. A semi-structured interview guide (Luo & Wildemuth, 2017) was constructed prior to the interviews. Both users and staff were asked questions regarding their experiences and perceptions of the revised CIP process and what it had led to for them. See Appendix 1 for the full interview guides.

### Analysis of collected data

Data was analysed following the six phases of reflexive thematic analysis (Braun & Clarke, 2022b): 1) familiarization with the data; 2) coding the data; 3) generating initial themes from the codes and coded data; 4) reviewing and developing themes; 5) defining, naming, and refining themes; and, 6) writing the report. Overall, in the present study, this was an iterative process of going back and forth between the phases. The analysis was also iterative between going back and forth between the manifest and latent content (Braun & Clarke, 2022b) of the interviews, in which the manifest involves statements from interviews and the latent involves the interpretated meaning of these statements. Phase one was conducted by the author conducting all interviews, transcribing some of them, and reading all transcripts several times. Thereafter, transcripts were interpretated and initial codes were produced. Codes were listed on a separate sheet of paper to keep track of the growing body of codes in a systematic way. Themes were thereafter constructed based on the codes. In the process of going back and forth between phases, themes were reviewed both based on the initial coding and in relation to the transcripts. A preliminary table with codes, sub-themes, themes, and an overarching main theme was constructed, and all transcripts were read again to check for coherence.

During discussions held with supervisors and reviewing of themes throughout the analysis, it also became clear that one theme with two sub-themes (a theme related to outcomes, with the sub-themes being user- and staff-related outcomes) overlapped other sub-themes and was integrated into these. A new table with themes and sub-themes was thereafter constructed based on the discussions and review of the themes. The table was reviewed to check for coherence with codes and data extracts. The iterative process of writing and reviewing also had the intention to ensure that each theme captured a shared meaning, not a plain summary of different topics, and that the themes were distinct. Therefore, some alterations to themes were made during the writing processes, illustrating how this is also an important part of the analysis. The author wrote the findings section, which was read by supervisors and was the basis for final discussions regarding the analysis and defining of themes. The themes were also given their final labelling during the discussions held.

The analysis of the collected data was data-driven, meaning that no categorization matrix based on the theoretical concepts was made prior to the analysis. However, after the completion of the analysis, the written material was linked to concepts related to epistemic (in)justice (Fricker, 2018) to more deeply explore how the experiences of users and staff can be understood.

### Reflexivity and trustworthiness

This study is linked to a constructionist paradigm, meaning that I recognize my active role in constructing themes and acknowledging my subjectivity in this process as a resource (Braun & Clarke, 2022a). This also means that the analysis is not labelled as inductive, because I bring myself, my knowledge, and my theoretical underpinnings into the analysis even though this has been data driven, or grounded in data (Braun & Clarke, 2022a). The emphasis on using preunderstanding as a resource in the analysis is also one of the reasons reflexive thematic analysis was chosen.

Braun and Clarke (2022a) emphasize the importance of reflexivity throughout the research process when conducting reflexive thematic analysis. This relates to both reflexive practices such as journaling but also being aware of one’s position in relation to the area of research and the participants. Reflexivity in the present study has been conducted with actions such as noting reflections and observations when reading transcripts, by constructing mind maps during the analysis process, and by engaging with their supervisors in discussions that focus on how the findings can be understood in a deeper sense. Braun and Clarke (2022a) describe that discussions when doing reflexive thematic analysis do not strive for validation or consensus of themes, but instead are a way of deepening the interpretations and understandings.

Strategies to ensure trustworthiness have been employed, alongside discussions with supervisors. These include triangulation of sources (staff and users), providing context to support transferability, documenting the research process, ensuring transparency in how data was collected and analysed, and presenting quotes to illustrate how interpretations were made, in alignment with the criteria for trustworthiness in thematic analysis as outlined by Nowell et al. (2017).

Regarding position, I am a social worker and have worked full time in social services with users and staff before the implementation of the revised CIP process. During the implementation process, I have worked at one of the sites for 1 month. Hence, I have practical experience and knowledge of the context. I also know staff at one of the sites, but I have intentionally not worked with any of the users involved in the study to avoid any potential power imbalance problems or role conflicts.

### Ethical considerations

The study was approved by the Swedish Ethical Committee (no. 2019–00657 and complement no. 2019–03971). In accordance with the ethical guidelines of the Swedish Research Council (n.d.) all participants were anonymous and were informed verbally and offered written information regarding participation. Each volunteer was told that their participation was voluntary and would not affect in any way the support they were given by social services (if they were users) or their employment (if they were staff), and that they could withdraw at any point without giving a reason. They were also told how their details were stored. They consented verbally on record. Additionally, the participants were offered the contact details of the author who conducted the interviews. Due to the low number of participants and a risk that individuals could be identified with overly specific quotes, all quotes were carefully considered and anonymized.

## Results

The results illustrate the experiences of users and staff when using SDM in the context of CIP. As shown in Table I below, the overarching main theme relates to a structured CIP process with user involvement that gives a feeling of safety and trust. These feelings of user involvement, safety, and trust are evident in all constructed themes.

**Table I.** An overview over themes and sub-themes.

A structured CIP process with user involvement that gives a feeling of safety and trust						
Themes	Emotional security through predictability			The importance of partnership		
Sub-themes	Having a predetermined structure	User involvement through preparations	Following through on expectations and commitments	Users being respected as contributors: the intention	Reaching a joint understanding	Continuity of care through staffs’ collaboration

### Emotional security through predictability

This theme captures the experiences of users and staff with the predetermined structure that is incorporated in a CIP process that involves SDM. The predetermined structure is experienced to give a feeling of emotional security by knowing what is going to happen. This structure relates to preparing the meeting together with the user, the CIP meeting, and having a subsequent follow-up meeting. Hence, this theme captures the more stringent meanings of a CIP process that involves SDM in terms of a structure and feelings connected to this structure.

#### Having a predetermined structure

Both users and staff showed that the predetermined structure enables user involvement throughout the revised CIP process due to a clear focus on the user’s expressed needs, wishes, and questions from the preparation to the follow-up meeting, illustrating how active participation (Nouf & Ineland, 2023) on behalf of the user is incorporated in the revised CIP process and how user knowledge is acknowledged. The new form for documentation was perceived by both users and staff to enable this predetermined structure with active user participation by giving users and staff something to hold on to in the CIP meeting and a plan to follow. None of the users saw any disadvantages with conducting CIP in this way. Rather, this increased structure was perceived by users and staff as one of the main benefits of conducting CIP in this new way:

But there’s a big difference, I think. It’s like more organized with everything. It’s superb.” (E, user)

Here it becomes easy that one can constantly redirect the focus to those goals. That you have talked about beforehand. And those goals are the ones you constantly go back to, like have we finished talking about this goal now, um okay then we move on to the next. It becomes more structured. And not as vague. As it can be otherwise. (K, staff)

The predetermined structure where users and staff meet each other over time was also experienced as important to establish mutual trust. The importance of mutual trust in relation to comorbidity was also illustrated. For example, one of the users described that people with a diagnosis can have “a lot of lack of trust” (C) and are hesitant with what information they share with others. Working together over time and getting to know each other as implied when utilizing the revised CIP process is seen by both users and staff as something that shapes mutual trust between them.

But I can experience it among some [staff], like, “have you taken” it’s very like that, you know. It can be a bit tough/ … /I think it is his trust in me that has increased, you know. But I feel he sees a difference. And he sees a difference when he meets me often. (B, user)

Users have stated in interviews how they needed the predetermined structure because it made it easy to understand the whole CIP process, allowed them to know what issues were on the meeting agenda, and enabled them to make sure that the correct issues would be discussed at the meeting. Users believed this was important due to their difficulties or control needs. It was also described by users and staff that meetings could be shorter due to having a structure and an agenda for the meeting, which made it easier to reach a joint decision. Users also illustrated that they had individual preferences regarding whether new topics could be raised at the CIP meeting or if the agenda needed to be followed strictly. Some topics are very personal, which is why staff need to ask the user before the meeting if they could be discussed, whilst flexibility was also seen as important. A benefit with the new form for documentation was also that relevant topics, pre-decided with the users, were raised during the meeting and that the meeting did not “sail away” (B) to focus on other things, which illustrated the need to follow the predetermined agenda for the CIP meeting.

It was also noticeable in interviews with users how the structure in the revised CIP process enabled them to have more structure in their lives overall. For example, one user described that getting a better structure made it easier for her to follow plans. Earlier in her life she missed meetings, but with this structure she was more structured herself and went to meetings. Another of the users described how the process with follow-up meetings and ongoing support led to a positive influence in her life.

Um, no, but I mean, I’m not… Overall… I mean, my life becomes more structured because of this [the revised CIP process], it has become. I mean, I became drug-free and got a job earlier this year. And I wouldn’t have been able to… I wouldn’t have been able to make it work if I hadn’t had this support, in this way. (B, user)

A predetermined structured plan and knowing what is expected also means that users do not have to worry about whether they are going to receive financial support. Both users and staff described how this has been a worrying process for the users because they did not know if they would receive benefits that are vital for their situation.

#### User involvement through preparations

The importance of the preparation was also highlighted in interviews with both users and staff. Overall, the advantage with the preparation also lays in increased user involvement in the whole process. Without the preparation and knowing what users want to discuss, it could be difficult to raise the user’s topics at the CIP meeting and the meeting could be viewed as chaotic. Staff described that this means that CIP will be the user’s plan, as intended, and not a forum for professionals to discuss their situations. This can increase user involvement in the CIP meeting, instead of the user becoming silent because none of their questions were raised, illustrating the active participation of the users (Nouf & Ineland, 2023) when utilizing the revised CIP process.

I think previously, or one probably used CIP a lot for, like… one’s own, now I have questions for healthcare one might have thought, but the user is barely involved in it, that you talk a bit over their head like that, it’s easy for that to happen. But I think with this approach, the user is involved from the beginning, which I think is very important. And I’ve had people say that too. (G, staff)

Staff described how the preparation helped them to prepare themselves and look up answers to the user’s questions before the meeting, enabling them to give answers in the CIP meeting. Hence, this revised CIP process enabled staff and users to come prepared to CIP meetings, which was perceived as important. The feeling of being prepared at a meeting was perceived by staff as a positive influence on their work.

In the interviews, it also became clear that the preparation makes it possible to invite the appropriate staff to the meeting, based on the agenda that the staff and users have decided on together. Instead of inviting everyone the users have contact with, they had to think about the role of everyone invited and why that person should participate in the CIP. For example, staff described how in the past they had been invited to CIP meetings without knowing why they were invited. The preparational phase of the revised CIP process can also lead to the establishment of trust.

But I think that with this preparatory phase, you gain more knowledge about, you can discuss what interventions are available, what do you have to offer, what can we offer, what’s best for the user. Um, in that discussion, I think that trust can be built. When you have time to sit together and address the issues. What does the person need? I think that can strengthen the trust in this process. (L, staff)

The preparation also enabled users to receive support from staff in the actual CIP meeting, if needed. None of the users interviewed said that they had a relative or friend with them in the meeting. It was observed by users that they can perceive these meetings as “difficult in general”, with several staff involved that had not met the user before the meeting. One of the users described that she felt that she should have had someone with her that she trusts, but she had no one who could. However, she felt that the social worker who had prepared the meeting together with her was on her side all through the meeting, which was good. Another user also described how the social worker who had prepared the meeting with her managed the meeting and involved her in the discussions.

But she had her notebook where she read out the questions and what I had arrived at. And she asked me, like, ‘was this what you wanted to bring up?’ That I got the voice, but she helped. (A, user)

#### Following through on expectations and commitments

In the interviews, different aspects of following through on expectations and commitments were illustrated. Following through on commitments was especially important in relation to establishing mutual trust between all involved in the CIP process, seeing that other people follow through on their commitments. This relates to both trust between users and staff, but also between staff from different units.

Many times there are expectations regarding what different professions, or what other professions should be able to offer. And, hopes, and it’s not always that one can come to an agreement so, it’s inevitable that it’s like that sometimes. But I think that one can also build more trust by, well, yeah, but we, for example, during follow-ups, especially I think that one does what one has said. And by it becoming this, team, well, a bit more of a team feeling, one can clarify more what expectations one can have of each other. (J, staff)

Moreover, the importance of having the follow-up meeting was expounded upon in interviews with both users and staff. The follow-up meeting was experienced by users and staff as enabling plans to be followed and resulting in definitive actions.

And I think it’s much better now, when there’s follow-up. Follow-up has been really, really important because otherwise things are just said and then it’s like nothing comes of it. (B, user)

One positive influence of utilizing the revised CIP is therefore that the CIP process leads somewhere. Overall, users described that this CIP process has helped them receive the support they need and get answers to their questions. Staff elaborated on the importance of this and stated that because of the follow-up meeting users had an ongoing process and that they did not have to wonder what was going to happen and what they should be doing. The process could also lead to a feeling of emotional security in that the users had knew what the plan was and the responsibility of everyone. Even though there might be a staff turnover, there was still a plan that could be followed, which was beneficial.

### The importance of partnership

This theme captures users and staff experiences of the importance of partnership between users and staff and also between staff from different units, as well as the significance of partnership in the CIP process that involves SDM. Hence, this theme illustrates the softer, relational meanings of a CIP process that involves SDM, such as staff’s approach to users and collaboration.

#### Users being respected as contributors: the intention

Both users and staff showed how SDM made users involved and equal partners in the CIP process, which could be understood as users being respected as contributors. However, it was also evident that this was the overall intention, even though it was not always the case in practice. For example, when utilizing the revised CIP process, users shared descriptions of *being listened to*. Users overall perceived that staff were quiet and listened to them, and that the meeting was calm, which illustrated how users were contributors in this CIP process.

Yeah, but I got to explain my situation and all that. They were very attentive and listened to me, no one interrupted or anything. I think maybe these kinds of conversations were much common before, it was much like that, I think. Not being able to have one’s say, you know. Now it was a much calmer meeting and all that. (E, user)

However, users also illustrated in interviews how they have experienced that a member of staff needed to validate their situation and needs to other staff for them to be taken seriously due to their comorbidity which could be understood as testimonial injustice because users were not being viewed as trustworthy knowledge bearers (Fricker, 2018). As the quote below illustrates, using the revised CIP process enables users to feel heard and to receive support from one unit in their contact with other units, strengthening their position in this context despite experiences of not being listened to.

But I think it’s moving forward slowly, it is. This approach helps a lot. There’s significant pressure on healthcare to take one seriously, you know. For mental health and physical health, and then addicts, it’s difficult to be heard in healthcare if you don’t have social services with you. (B, user)

The documentation of staff throughout the revised CIP process is viewed by users as an indication that the staff are listening and taking seriously what the users say. Users also described in interviews that staff validated their perceptions of the user’s situation with the user by reading the documentation out loud and asking if they had understood their situation correctly. This made the user feel understood during the CIP process, and that staff wanted to understand their situation. Staff validating the user’s situation with the user directly instead of with other staff can be understood as staff trusting the users and viewing them as credible epistemic subjects (Fricker, 2018; Nouf & Ineland, 2023). It was also described that users felt important and felt that the CIP process was something staff wanted to do, not something that they must do.

Also, staff shared stories regarding how users were more included and an equal part in the revised CIP-process. It was also described how it can be strengthening for the user to be involved like this in a process. Staff also described how the process could enable the person to feel more engagement themselves and have the will to change their situation. One of the main benefits of this process described by staff is the users being more involved. Users were more involved in that they were listened to and that they shared their views of their situation, but also that the user needed to reflect more upon their situation overall. In this way, users could become epistemic subjects and active participants (Nouf & Ineland, 2023) contributing to the discussions in the CIP process. However, this was also viewed as a challenge by staff who said that users were not used to being asked about their needs, which was why some users at first could be quite hesitant towards answering this question.

Staff also reflected upon how users being contributors could lead to an increased trust between staff and users.

That it feels more professional now than before. And that they can build trust. Okay, they have listened to me. They have gone through my case. And know what problems I have. They have listened. They have taken me seriously, like umm. I think that can strengthen the trust. (L, staff)

Overall, users contributing with their perspectives on what support they needed was perceived as something positive by staff. For example, it was described how this did not necessarily mean that the users would receive the specific support they wanted, but that it was something that could be the basis of the ongoing discussions. These aspects were, however, problematized because it could be a disadvantage if the users felt that the support they wanted was something they were going to get and that they were not open to alternatives. This illustrated how staff, even though utilizing SDM, can be unsure about the active participation of the users (Nouf & Ineland, 2023) throughout the CIP process, and how to handle situations when their perspectives were not aligned.

#### Reaching a joint understanding

This sub-theme relates to reaching a joint understanding of the user’s situation through collaboration between the user and staff over time. Both users and staff illustrated this sub-theme in interviews in that they through discussions reached a joint understanding of the user’s situation. This was deemed important because users at times were unsure how staff perceived their situation and because there could be misunderstandings or different perspectives that needed to be discussed and cleared. For example, one user described that it was “a little shock” to hear how her social worker had understood her situation and how they had completely different views of her situation. However, the user was unsure whether she had understood the social worker correctly at first, but they could discuss her situation together and resolve any misunderstandings. This could be understood as active participation of the user (Nouf & Ineland, 2023) facilitating a joint understanding of the user’s situation.

Staff also described how joint discussions regarding the user’s situation gave them knowledge regarding the user’s perceptions. With the new way of conducting CIP, they could clarify what the user’s perceptions were and put these in the centre to establish a joint understanding of the user’s situation based on the user’s perspectives.

That often it’s from our perspective, like, “this is what you need help with.” And that’s what I think the difference is in this././Returning the ball to where it should be. Because it might be something that, well, I think we still have, I mean, professionals’ perspective on the needs. Because we can still bring that in. If there’s something that we perceive, that the user might not. (I, staff)

As illustrated in the quote, staff said that they could still state their views even though they were more inclined to also ask the users about their views. Staff also described how these discussions of the user’s situation carried on throughout the CIP process. For example, in the CIP meeting, both users and staff shared their perspectives regarding every item on the agenda, demonstrating active participation (Nouf & Ineland, 2023) in the CIP meeting.

#### Continuity of care through staffs’ collaboration

In interviews, it became clear that users and staff had experienced continuity of care in collaboration with the staff throughout the CIP process. This was perceived as positive by both users and staff. For example, one member of staff described how she felt proud conducting the revised CIP process because it turned out well and she received positive feedback from other staff involved. A member of staff also stated that work became more fun, and she saw more quality in her work when conducting CIP in this new way with collaboration over time. In interviews with both users and staff it was also described that a benefit with the revised CIP process was that the structure also improves collaboration between staff.

So, I think it’s been better now because it’s been more clear. And I’ve, like, how should I say, it felt more serious. (C, user)

This structure also relates to having the same form for documentation and working in the same way, which according to staff this revised CIP process has facilitated. This also means that staff *understand each other*, which is good for collaboration. One member of staff also elaborated on how continuity with working in the new way can lead to a feeling of more quality in the CIP meetings and a benefit in collaborating in this new way.

I believe that, and I think over time, people will experience that there’s more quality in these meetings, and maybe they also see a benefit in collaborating in this way. From all perspectives. That collectively, we help these individuals become healthy and well, and that’s what we all want, you know (G, staff)

Users overall described how CIP is important for them and how staff talking with each other makes it easier for the users because they do not have to call several members of staff to coordinate their own support. Even though this can mean that several staff members participate in the same meeting, the benefit is that the users do not have to call them one by one.

Users also shared descriptions of staff talking with each other in the preparation when the user was not present. Overall, this was deemed important because this lessened the burden for the user in coordinating the support on their own. It also made it easier for staff to develop an understanding of their situation and come prepared to the CIP meeting. However, it was also deemed important that staff did not talk about everything without the user present. One of the users, for example, described that she thought it was difficult before when staff talked about her, and she was wondering what they were saying. However, now she believes it is good because it indicates that staff are thinking about her and wants what is best for her, illustrating how users’ trust in staff enables the preparational part of the CIP meeting.

Overall, it was also described that it is *better when staff have talked with each other* when preparing for the CIP meeting since more uncertainty when this was not done was illustrated

It feels more like I know what to expect, of course, when I have, talked with the other parties involved beforehand. Then I also get a sense of which people will be there. There are no uncertainties, um, unlike when I haven’t done that, then it can be a bit like, who is it from there, from the social services unit, I didn’t have the name of the caseworker, just wrote responsible caseworker. Who is it coming there, could it be that it’s a mistake that they’re not coming now? (J, staff)

However, one member of staff also stated that CIP is not always better even though the preparations are made because the invited staff can question the need for a CIP or do not understand their role in the meeting because they have nothing to contribute. This illustrated the importance of staff trusting each other’s knowledge and reasons for inviting each other to a CIP meeting. Nevertheless, staff stated that practical issues could be sorted out in the preparations if staff talked with each other before the CIP meeting. For example, they have experienced other staff not coming to a CIP meeting they have been invited to. However, having the preparational contact means that they can decide upon a time together. One member of staff also described how the motivation to go to a CIP meeting increased if one has been a part of the whole process.

So, I think you might also become more motivated as staff to attend, I believe. Um, especially if you’ve had the chance to call and say that I’m considering a CIP and I’d like to add something, and does this date work for you. Unlike when the invitation just show up in the mailbox that here, you have a CIP here, um, then I think the staff might become more motivated perhaps. (K, staff)

According to staff, being prepared for the meeting can also lead to calmer meetings where no one must “enter defence mode” because everyone knows what the meeting is about.

## Discussion

This study has explored the experiences of users and staff of utilizing a revised CIP process that incorporates SDM. Overall, the participants experienced a structured CIP process that fostered a sense of safety and trust. This relates to the more stringent meanings of having a predetermined structure with user involvement, as well as the importance of partnership, both of which are facilitated through this revised CIP process. Overall, the result provides insights that conducting CIP in this new manner strengthens the users’ position as knowledge contributors in the CIP process. The preparational part of a CIP process that involves SDM ensures user participation and structure throughout the process, whilst the follow-up is important to enable mutual trust between users and staff and between staff from different organizations. Hence, the revised CIP process is experienced as enhancing user participation, structure, and mutual trust.

Previous research within the mental health field (Grim et al., 2019; Kurs & Grinshpoon, 2018; Nouf & Ineland, 2023) illustrates users having experiences of not being heard or trusted in encounters with staff. As put forward in this previous research, this is a form of epistemic injustice (Fricker, 2018) because users are not viewed upon as capable and credible epistemic subjects. Findings in this article illustrate experiences of other staff needing to validate the user’s stories to enable receiving care. Through the lens of epistemic injustice, this can be understood as the user’s knowledge being devalued in relation to staff knowledge, which is also evident in previous research (Wenaas et al., 2021).

However, the results also illustrate how there is a shift towards the users when using the revised CIP process. This shift involves users being a part of discussions and involved in the decision-making process in a way where they can initiate discussions regarding different treatment options and object to proposals of the staff or to their understanding of their situation. This is a form of active participation (Nouf & Ineland, 2023) that is emphasized in guidelines and policies. Integrating SDM into the CIP process therefore possesses the possibility to utilize user participation as intended when CIP was legislated in 2010. Moreover, this form of active participation means that users are valued as contributors to the shared understanding and decisions made throughout the CIP process, in contrast to epistemic silencing (Hookway, 2010) or passive participation (Nouf & Ineland, 2023).

Epistemic silencing could lead to instances where users internalize others’ views, which prevents them from active participation in their treatment plans due to low confidence. This prevention from active participation could lead to reduced adherence to their treatment (Kurs & Grinshpoon, 2018). In the present study, it was evident that the revised CIP process influenced users following plans, coming to meetings, and having more structure in their lives when being in partnership with staff when establishing their treatment plans. These findings illustrate the importance of active participation and epistemic justice in decision-making processes.

Users in the present study have related feelings of being heard and taken seriously when partaking in the revised CIP process, whilst staff illustrated how users were placed in the centre and perceived as partners. Moreover, the results showed that the preparational discussions gave a shared understanding of the user’s situation. In other words, utilizing the revised CIP process could ensure testimonial justice (Fricker, 2018) in which user’s perspectives were not dismissed. Rather, they were the basis for a shared understanding of their situation and for discussions held through the CIP process. However, it was also stated that it was important to receive staff’s perceptions of the user’s situation to establish this joint understanding. It has been shown in previous research that shared understanding between users and staff and between different groups of staff regarding, for example, realistic goals could mean that the plan is useful because it became a tool to use in practice (Beck et al., 2021).

The importance of mutual trust in relation to user participation has been shown in previous research (Jones et al., 2021). In the present study, the revised CIP process was seen to contribute to this enhanced mutual trust. Besides mutual trust between users and staff, the results also indicated that the revised CIP process could enhance trust between staff, e.g., deciding upon something and other staff following through on their commitments to the follow-up meeting enhanced trust between staff. In previous research it was shown that trust itself is an important facilitator of collaboration between staff (Andersson et al., 2011).

Even though it was stated that the innovation increased user participation in the decision-making process, this was mostly related to who to invite, what should be brought up in the CIP meeting, what goals should be set in the preparation, strengthening the users position in this context. However, findings from the study focusing on staff perspectives of the implementation process (Jones et al., in manuscript) illustrated that there persisted some difficulties with user involvement in the actual decision-making, such as limitations to what degree users could decide upon support because of available support or resources. Also, findings in the present study revealed difficulties when the perspectives of staff and users regarding items such as treatment options did not align. Hence, the results illustrate that user involvement is enhanced in the CIP process whilst there prevail questions regarding their involvement in the actual decisions. This also relates to organizational rationales brought up by Björk (2019) and that users and staff in social work are not autonomous in the decision-making. Rather the decision-making process is also influenced by organizational rationales such as financial resources.

### Limitations and future research

An overall limitation in the present study is the lack of knowing how the revised CIP process was used since fidelity has not been explored deeper through for example observations. Hence, it is uncertain if the steps of incorporating SDM was utilized as intended throughout the whole process; or if the innovation encouraged CIP to be utilized as intended in legislation with preparation and follow-up. Nevertheless, users and staff experienced changes in how CIP was conducted, especially due to the discussions over time that contributed to a shared understanding of the user’s situation, which is in alignment with SDM.

A limitation with convenience sampling is the challenge with controlling for representatives (Wildemuth, 2017). However, having a representative sample is not the overall intention with qualitative research (Polit & Beck, 2021). In qualitative research, the intention instead is to obtain diverse participants representing a variety of experiences of the phenomenon being explored. Convenience sampling sets a limitation to this because it does not ensure that the participants are the ones that can provide the most rich information and different descriptions of the phenomena (Polit & Beck, 2021). Hence, convenience sampling does not ensure a broad representation of experiences. This study shows positive experiences of utilizing SDM in CIP, and contradicting experiences are scarce, which illustrates a lack of variety in experiences.

Both selection bias and needing to rely on others to invite people to a study influence studies (Patterson et al., 2010). For example, Patterson et al. (2010) has illustrated that a poor relationship between a therapist and patient lessens the likelihood of the patient being referred to a research team. This could be the case in the present study because all users involved had a good relationship with their social worker, even though this was not always the case. Future research would therefore benefit from including users in the preparation and conducting of studies within this field to jointly determine how to reach out with information about the study and recruit participants without depending solely on staff.

Regarding staff, those who participated were the ones who tried the innovation. Overall, we know from the study focusing on staff perspectives of the implementation process (Jones et al., in manuscript) that there was a variety regarding if and how the innovation was used. This could influence the results. For example, staff who tried the innovation could be viewed upon as champions in that they are more eager to try new things, and therefore more positive than staff who did not try the innovation.

Most participants in the study were female (all users), which also exemplifies a challenge with convenience sampling. However, Thomas et al. (2021) and Fisher et al. (2021) illustrates in systematic reviews that most participants in previous studies regarding SDM involved males, so this study contributes with knowledge from a less researched perspective.

An unexpected finding in the present study was the influence of SDM in the users’ lives overall, such as structure, work, and sobriety. This would be of interest to explore deeper in future research, focusing more on experiences of the influence of SDM in the personal life with more participants to gain a larger understanding.

## Conclusions

These findings suggest that the revised CIP process that involves SDM can contribute to shifting staff perspectives of users as capable and credible knowledge bearers in CIP and enhance collaboration between staff from different caregivers with enhanced mutual trust between all involved parties in the decision-making process.

## Data Availability

The data supporting the findings of this study are not publicly available due to ethical considerations. Participants were assured of confidentiality, and consent to share the raw data was not obtained. Therefore, to protect the privacy and confidentiality of the participants, the data cannot be accessed by readers.

## Appendix 1. Interview guides

User interviews

Have you participated in a CIP-process prior to this one?

If so – did you notice any differences? What differences were there?

Describe the CIP-process that you have participated in

How was the preparation conducted?

How was the meeting conducted?

How was the follow-up conducted?

How did you experience this CIP-process?

What were the advantages of this CIP-process?

What were the disadvantages of this CIP-process?

How do you perceive your participation in this CIP-process?

Have you been listened to, felt heard, involved in the setting of agenda?

How do you perceive your knowledge in the CIP-process?

Have your knowledge been used? How?

How do you perceive the collaboration between social services and healthcare?

Do you perceive that this CIP-process has led somewhere?

How? To what? Made plans regarding treatment, support?

Has this CIP-process influenced trust between you and providers? How?

Anything you like to add?

Staff interviews

How did you experience this CIP-process?

How do you perceive user participation in conjunction with CIP now, compared to before?

How is user participation influenced- if at all?

How do you perceive user participation in the different steps?

How do you perceive the user’s role in conjunction with CIP now, compared to previously?

How do you perceive collaboration with other staff?

Is there any difference from before?

How do you perceive trust between staff and users in this CIP-process?

Is there any difference from before?

How do you perceive trust between staff and users in this CIP-process?

Is there any difference from before?

Anything you like to add?

## References

[cit0001] Adams, N., & Grieder, D. M. (2014). *Treatment planning for person-centered care: Shared decision making for whole health*. Elsevier Academic Press.

[cit0002] Andersson, J., Ahgren, B., Axelsson, S. B., Eriksson, A., & Axelsson, R. (2011). Organizational approaches to collaboration in vocational rehabilitation − an international literature review. *International Journal of Integrated Care*, 11(4), e137. 10.5334/ijic.670 22128280 PMC3225240

[cit0003] Askheim, O. P., Christensen, K., Fluge, S., & Guldvik, I. (2017). User participation in the Norwegian welfare context: An analysis of policy discourses. *Journal of Social Policy*, 46(3), 583–14. 10.1017/S0047279416000817

[cit0004] Beck, A.-M. T., Rasmussen, B. M., & Nielsen, T. K. H. (2021). Action plans as active boundary objects. *Research on Social Work Practice*, 31(4), 382–389. 10.1177/10497315211002637

[cit0005] Beresford, P., & Croft, S. (2004). Service users and practitioners reunited: The key component for social work reform. *British Journal of Social Work*, 34(1), 53–68. 10.1093/bjsw/bch005

[cit0006] Björk, A. (2019). Reconsidering critical appraisal in social work: Choice, care and organization in real-time treatment decisions. *Nordic Social Work Research*, 9(1), 42–54.

[cit0007] Braun, V., & Clarke, V. (2022a). Conceptual and design thinking for thematic analysis. *Qualitative Psychology*, 9(1), 3. 10.1037/qup0000196

[cit0008] Braun, V., & Clarke, V. (2022b). *Thematic analysis: A practical guide*. SAGE.

[cit0009] Chmielowska, M., Zisman-Ilani, Y., Saunders, R., & Pilling, S. (2023). Trends, challenges, and priorities for shared decision making in mental health: The first umbrella review. *The International Journal of Social Psychiatry*, 69(4), 823–840. 10.1177/00207640221140291 36680367 PMC10240653

[cit0010] Elwyn, G., Frosch, D., Thomson, R., Joseph-Williams, N., Lloyd, A., Kinnersley, P., Cording, E., Tomson, D., Dodd, C., Rollnick, S., Edwards, A., & Barry, M. (2012). Shared decision making: A model for clinical practice. *Journal of General Internal Medicine*, 27(10), 1361–1367. 10.1007/s11606-012-2077-6 22618581 PMC3445676

[cit0011] Eriksson, E. (2015). *Sanktionerat motstånd: Brukarinflytande som fenomen och praktik*. Diss. Lund: Lunds universitet.

[cit0012] Fisher, A., Mills, K., Teesson, M., & Marel, C. (2021). Shared decision-making among people with problematic alcohol/other drug use and co-occurring mental health conditions: A systematic review. *Drug & Alcohol Review*, 40(2), 307–324. 10.1111/dar.13149 32902078

[cit0013] Fricker, M. (2018). *Epistemisk orättvisa: Kunskap, makt och etik (C.-F. Brück, Trans.)*. Thales.

[cit0014] Gibson, A., Cooper, M., Rae, J., & Hayes, J. (2020). Clients’ experiences of shared decision making in an integrative psychotherapy for depression. *Journal of Evaluation in Clinical Practice*, 26(2), 559–568. 10.1111/jep.13320 31788932

[cit0015] Grim, K., Tistad, M., Schön, U.-K., & Rosenberg, D. (2019). The legitimacy of user knowledge in decision-making processes in mental health care: An analysis of epistemic injustice. *Journal of Psychosocial Rehabilitation and Mental Health*, 6(2), 157–173. 10.1007/s40737-019-00145-9

[cit0016] Hamann, J., & Heres, S. (2019). Why and how family caregivers should participate in shared decision making in mental health. *Psychiatric Services*, 70(5), 418–421. 10.1176/appi.ps.201800362 30784381

[cit0017] Haugom, E. W., Stensrud, B., Beston, G., Ruud, T., & Landheim, A. S. (2020). Mental health professionals’ experiences with shared decision-making for patients with psychotic disorders: A qualitative study. *BMC Health Services Research*, 20(1), 1093. 10.1186/s12913-020-05949-1 33246451 PMC7694931

[cit0018] Hookway, C. (2010). Some varieties of epistemic injustice: Reflections on fricker. *Episteme*, 7(2), 151–163. 10.3366/epi.2010.0005

[cit0019] Jones, A., Jess, K., & Schön, U. K. (2021). How do users with comorbidity perceive participation in social services? A qualitative interview study. *International Journal of Qualitative Studies on Health and Well-Being*, 16(1), 1901468. 10.1080/17482631.2021.1901468 33752576 PMC8725697

[cit0020] Knutsson, O., & Schön, U.-K. (2020). Co-creating a process of user involvement and shared decision-making in coordinated care planning with users and caregivers in social services. *International Journal of Qualitative Studies on Health and Well-Being*, 15(1), 1812270. 10.1080/17482631.2020.1812270 32940581 PMC7534304

[cit0021] Kurs, R., & Grinshpoon, A. (2018). Vulnerability of individuals with mental disorders to epistemic injustice in both clinical and social domains. *Ethics & Behavior*, 28(4), 336–346. 10.1080/10508422.2017.1365302

[cit0022] Laitila, M., Nikkonen, M., & Pietilä, A.-M. (2011). Involvement in mental health and substance abuse work: Conceptions of service users. *Nursing Research & Practice*, 2011, 1–8. 10.1155/2011/672474 PMC3169363 21994839

[cit0023] Lukens, J. M., Solomon, P., & Sorenson, S. B. (2013). Shared decision making for clients with mental illness: A randomized factorial survey. *Research on Social Work Practice*, 23(6), 694–705. 10.1177/1049731513489734

[cit0024] Luo, L., & Wildemuth, B. M. (2017). Semistructured interviews. In B. M. Wildemuth (Ed.), *Applications of social research methods to questions in information and library science* (Vol. 2, pp. 249–257). Libraries Unlimited.

[cit0025] Matscheck, D., & Piuva, K. (2022). Integrated care for individuals with mental illness and substance abuse - the example of the coordinated individual plan in Sweden. *European Journal of Social Work*, 25(2), 341–354. 10.1080/13691457.2020.1843409

[cit0026] Matscheck, D., Piuva, K., Eriksson, L., & Åberg, M. (2019). The coordinated individual plan - is this a solution for complex organizations to handle complex needs? *Nordic Social Work Research*, 9(1), 55–71. 10.1080/2156857X.2018.1489886

[cit0027] Nouf, F., & Ineland, J. (2023). Epistemic citizenship under structural siege: A meta-analysis drawing on 544 voices of service user experiences in Nordic mental health services [review]. *Frontiers in Psychiatry*, 14. 10.3389/fpsyt.2023.1156835 PMC10272743 37333919

[cit0028] Nowell, L. S., Norris, J. M., White, D. E., & Moules, N. J. (2017). Thematic analysis: Striving to meet the trustworthiness criteria. *International Journal of Qualitative Methods*, 16(1). 10.1177/1609406917733847

[cit0029] Nykänen, P. (2020). Shared decision making in the social services? Reasons to consider when choosing methods for service user participation. *Journal of Evaluation in Clinical Practice*, 26(2), 569–574. 10.1111/jep.13323 31793157 PMC7155111

[cit0030] Nykänen, P., Schön, U.-K., & Björk, A. (2023). Shared decision making in social services – some remaining questions. *Nordic Social Work Research*, 13(1), 107–118. 10.1080/2156857X.2021.1958908

[cit0031] Patterson, S., Kramo, K., Soteriou, T., & Crawford, M. J. (2010). The great divide: A qualitative investigation of factors influencing researcher access to potential randomised controlled trial participants in mental health settings. *Journal of Mental Health*, 19(6), 532–541. 10.3109/09638237.2010.520367 21121823

[cit0032] Polit, D. F., & Beck, C. T. (2021). *Nursing research: Generating and assessing evidence for nursing practice*. Wolters Kluwer.

[cit0033] Rosenberg, D., Schön, U.-K., Nyholm, M., Grim, K., & Svedberg, P. (2017). Shared decision making in Swedish community mental health services - an evaluation of three self-reporting instruments. *Journal of Mental Health*, 26(2), 142–149. 10.1080/09638237.2016.1207223 27452763

[cit0034] Schön, U.-K., Grim, K., Wallin, L., Rosenberg, D., & Svedberg, P. (2018). Psychiatric service staff perceptions of implementing a shared decision-making tool: A process evaluation study. *International Journal of Qualitative Studies on Health and Well-Being*, 13(1), 1421352. 10.1080/17482631.2017.1421352 29405889 PMC5804774

[cit0035] Thomas, E. C., Ben-David, S., Treichler, E., Roth, S., Dixon, L. B., Salzer, M., & Zisman- Ilani, Y. (2021). A systematic review of shared decision-making interventions for service users with serious mental illnesses: State of the science and future directions. *Psychiatric Services*, 72(11), 1288–1300. 10.1176/appi.ps.202000429 34369801 PMC8570969

[cit0036] The Union for Professionals. (2017). *Ethics in social work. A code of conduct and ethical behaviour for social workers*. Akademikerförbundet SSR.

[cit0037] Vedung, E., & Dahlberg, M. (2013). *Demokrati och brukarutvärdering*. Studentlitteratur.

[cit0038] Wenaas, M., Andersson, H. W., Kiik, R., & Juberg, A. (2021). User involvement in interprofessional team meetings within services for substance use disorders. *Nordic Studies on Alcohol & Drugs*, 38(2), 190–203. 10.1177/1455072520978353 PMC8899073 35310002

[cit0039] Wildemuth, B. M. (2017). *Applications of social research methods to questions in information and library science*. Libraries Unlimited.

